# Cyber-Internet Security Framework to Conquer Energy-Related Attacks on the Internet of Things with Machine Learning Techniques

**DOI:** 10.1155/2022/8803586

**Published:** 2022-09-29

**Authors:** Anand Kumar, Dharmesh Dhabliya, Pankaj Agarwal, Nagender Aneja, Pankaj Dadheech, Sajjad Shaukat Jamal, Owusu Agyeman Antwi

**Affiliations:** ^1^Department of Computer Science and Engineering, Cambridge Institute of Technology, North Campus, Bangalore, Karnataka, India; ^2^Department of Information Technology, Vishwakarma Institute of Information Technology, Pune, Maharashtra, India; ^3^School of Engineering and Technology, K. R. Manglam University, Gurugram, Haryana, India; ^4^School of Digital Science, Universiti Brunei Darussalam, Bandar Seri Begawan, Brunei Darussalam; ^5^Department of Computer Science & Engineering, Swami Keshvanand Institute of Technology, Management & Gramothan (SKIT), Jagatpura, Jaipur, Rajasthan, India; ^6^Department of Mathematics, College of Sciences, King Khalid University, Abha, Saudi Arabia; ^7^Ghana Communication Technology University, Department of Telecommunications Engineering, Accra, Ghana

## Abstract

The Internet of Things (IoT) ushers in a new era of communication that depends on a broad range of things and many types of communication technologies to share information. This new age of communication will be characterised by the following characteristics: Because all of the IoT's objects are connected to one another and because they function in environments that are not protected, it poses a significantly greater number of issues, constraints, and challenges than do traditional computing systems. This is due to the fact that traditional computing systems do not have as many interconnected components. Because of this, it is imperative that security be prioritised in a new approach, which is not something that is currently present in conventional computer systems. The Wireless Sensor Network, often known as WSN, and the Mobile Ad hoc Network are two technologies that play significant roles in the process of building an Internet of Things system. These technologies are used in a wide variety of activities, including sensing, environmental monitoring, data collecting, heterogeneous communication techniques, and data processing, amongst others. Because it incorporates characteristics of both MANET and WSN, IoT is susceptible to the same kinds of security issues that affect those other networks. An assault known as a Delegate Entity Attack (DEA) is a subclass of an attack known as a Denial of Service (DoS). The attacker sends an unacceptable number of control packets that have the appearance of being authentic. DoS assaults may take many different forms, and one of those kinds is an SD attack. Because of this, it is far more difficult to recognise this form of attack than a simple one that depletes the battery's capacity. One of the other key challenges that arise in a network during an SD attack is that there is the need to enhance energy management and prolong the lifespan of IoT nodes. This is one of the other significant issues that arise in a network when an SD attack is occurs. It is recommended that you make use of a Random Number Generator with Hierarchical Intrusion Detection System, abbreviated as RNGHID for short. The ecosystem of the Internet of Things is likely to be segmented into a great number of separate sectors and clusters. The HIPS system has been partitioned into two entities, which are referred to as the Delegate Entity (DE) and the Pivotal Entity, in order to identify any nodes in the network that are behaving in an abnormal manner. These entities are known, respectively, as the Delegate Entity and the Pivotal Entity (PE). Once the anomalies have been identified, it will be possible to pinpoint the area of the SD attack torture and the damaging activities that have been taken place. A warning message, generated by the Malicious Node Alert System (MNAS), is broadcast across the network in order to inform the other nodes that the network is under attack. This message classifies the various sorts of attacks based on the results of an algorithm that employs machine learning. The proposed protocol displays various desired properties, such as the capacity to conduct indivisible authentication, rapid authentication, and minimum overhead in both transmission and storage. These are only a few of the desirable attributes.

## 1. Introduction

Wireless sensor networks have attracted an extraordinary amount of attention from the scientific community over the course of the last several years. A typical wireless sensor network is made up of thousands of sensor nodes that are scattered around an area of interest at random or according to a defined statistical distribution [1]. This may be done in accordance with a statistical distribution that has been specified. These nodes are distinguishable from others in the network by their low price, minuscule size, and limited energy capacity, which is often satisfied by a battery. In addition, their low cost and minuscule size make them easy to deploy. Tiny sensor nodes that are multifunctional are capable of detecting their surroundings, processing the data that they acquire, and communicating with one another through built-in antennae and RF waves. They are also capable of sensing the data that they collect from one another. In most cases, these networks are used to monitor a field of interest network in order to identify movement, changes in temperature and precipitation, and other phenomena of a similar kind [2]. It is only feasible for a single sensor node to observe a tiny portion of the surrounding environment because of the considerable resource restrictions of the node. On the other hand, when multiple sensor nodes collaborated together, they were able to carry out a far more extensive task in a way that was more efficient [3]. When compared to a wired sensor network, a wireless sensor network has several advantages, the most important of which are the low cost of deployment and the absence of the need for a tangled web of cables serving as the communication backbone [4]. There are situations when using wired communication backbones is either not practicable or is not economically comfortable. Wireless sensor networks make it possible for a wide variety of applications [5, 6], beginning with security surveillance in the military and on battlefields and extending all the way to the monitoring of environmental phenomena that had never been observed before, smart homes and offices, improved healthcare, industrial diagnosis, and a great deal more [5]. Wireless sensor networks enable a variety of applications [5, 6]. For instance, a sensor network could be set up on a remote island for the purpose of observing wildlife habitat and animal behaviour, or it could be placed close to the crater of a volcano in order to collect data on temperature, pressure, and seismic activity [7]. Both of these scenarios would serve the same purpose of gathering information. When it comes to many of these applications, the environment may be dangerous, which means that the engagement of humans is not an option. As a consequence of this, the sensor nodes will be positioned in a haphazard manner or scattered in the air, and they will remain unattended for a period of time ranging from months to years without having their batteries replaced [8]. Therefore, the management of resources, including energy, is of paramount importance to these networks [9]. One of the most active areas of research relating to wireless sensor networks right now is the study of coverage [10]. The degree to which a sensor network is able to provide a sufficient level of monitoring across a certain network region of interest is referred to as the network's “coverage.” To put it another way, you may think of it as an assessment of the entire quality of the service [11]. Depending on the circumstances, there are a number of different criteria that may be used in order to assess coverage. The capacity of a sensor network to maintain its connections is just as important as the network's capacity to provide coverage. Connectivity may be defined in a number of ways, one of which is the capability of the sensor nodes to communicate with the data sink. The data that were obtained by a sensor node will not be processed if there is no route available between that sensor node and a data sink [12]. Each node has a communication range that specifies the minimum distance between it and another node that must be maintained for it to be able to receive data from that other node. This is separate from the sensing range, which is what establishes the boundaries of the area that a node is able to keep an eye on [13]. Although the two ranges could be comparable in size, the behaviours that they display are often highly different from one another even if they might have some similarities. As part of the continuing research project, a network of wireless sensors is now being constructed. Each sensor node in this network will have the capacity to communicate in addition to sensing their surroundings. When it comes to a wireless sensor network, coverage and connectivity are two of the most important requirements that must be met. A network like this one is designed to either collect data or recognise events of interest, both of which are then sent to a fusion centre together with the information that was obtained from the various sources. Because of this, having a link, which is also known as the ability to transmit information to the fusion centre, is just as important as having sufficient sensing coverage. Therefore, the research that is now being conducted takes into account the quality of connection that is present in sensor networks. Massive sensor networks are the primary focus of our attention right now. As a consequence of advancements in Micro Electro Mechanical Systems (MEMS) [9] and wireless network technology, the use of teeny sensor nodes, sometimes commonly referred to as motes, has become a viable option. These are very small devices with limited coverage that have very little power, memory sizes that are less than typical, and bandwidth that is lower than average. The cost of these devices is very cheap. Wireless sensor networks comprise a large number of sensor nodes, and they are able to gather and distribute data in areas that are unsuitable for conventional networks due to environmental and/or strategic factors. Wireless sensor networks are also able to collect data in areas that are difficult to access. After this, they have a bright future in many different areas, including but not limited to: smart homes, smart farms, smart parking, smart hospitals, monitoring of habitats, buildings, and structures, distributed robotics, industry, manufacturing, national security, and many others. In recent years, wireless sensor networks have become more practical because of the falling cost of sensors. Because of this, wireless sensor networks have become more attractive as potential low-cost solutions to a broad variety of challenges that are experienced in real life. Even while every network is vulnerable to the same kinds of threats [14], remote wireless sensor networks are more vulnerable to security breaches [15] because it is physically simpler for prospective adversaries to get access to them. When it comes to the implementation of traditional security solutions, one of the most important challenges that might be presented is brought about by the memory and energy limits of sensor nodes. The fact that wireless sensor networks employ unreliable communication means, in addition to the fact that these networks are often unattended once they have been constructed, makes it exceedingly difficult to offer adequate security countermeasures [16]. Therefore, previous research has shown that the future of sensor nodes will lie in lowering the cost rather than growing the memory or energy capabilities. This is because cost reduction is easier to accomplish. The following factors contributed to the formation of this conclusion: 1976 research conducted by [17] Whitfield Diffie and Martin Hellman demonstrated the existence of a secure key exchange protocol already present in the network at that time. This technique enabled two users to exchange a symmetric secret key without disclosing any past secrets, whether they were communicating via an unsecured wired or wireless connection. The presence of a secure key exchange mechanism allowed for the accomplishment of this goal. Even in this day and age, the Diffie–Hellman protocol is one that continues to see widespread use and is well-known [18]. In spite of how simple it is to use, it might be vulnerable to a broad number of attacks [19], such as the man in the middle attack (MIMA) and others. The Diffie–Hellman key exchange system, like other cryptographic protocols before it, was very difficult to decrypt and needed a considerable amount of time to locate. The programmer of the Diffie–Hellman algorithm does not have sufficient awareness of the security problems, and as a result, the method is vulnerable to a broad range of attacks, including MIMA. Encrypting the prime random numbers before transmitting a key back and forth between two distinct parties is one of the steps that we take in the work that we have outlined in this article to ensure that the prime random numbers remain private. It is impossible to transmit any sort of data from one node to another without the presence of communication, which is an extremely important source that must be present. In order to achieve enhanced performance and get acceptability from users and client organisations, data sharing has to have a high degree of both quality and security [20]. When it comes to cryptography, maintaining the confidentiality of key distribution is one of the most important considerations.

In order to investigate the possibility of integrating the Internet of Things, smart environments need communication technologies and networks that are compatible with one another and can function together seamlessly. The bulk of the Internet of Things' capabilities are dependent on wireless sensor networks (WSN) since the Internet of Things is built up of many different sensors on the edge computing layer. MANET and WSN are both self-organised and multi-hop networks, therefore there are some parallels between the two [21]. On the other side, there are some differences between the two. In addition, since mobile nodes are what constitute a MANET, the topology of the network is almost always in a state of change. This is because mobile nodes may move around. In a wireless sensor network (WSN), the sensor nodes are responsible for choosing the right routing protocols. This is necessary because sensor nodes are needed to save their energy while the data are being delivered, as well as to reduce the amount of time spent processing the data. As a direct consequence of this, there is a continuous need for the convergence of MANET, WSN, and the Internet of Things.

The standards that must be met for a high degree of security in wireless sensor networks (WSNs) are, for the most part, equivalent to those that must be met for the security of any other kind of communication system. The availability, authenticity, integrity, and secrecy of the information are some of the characteristics that must be met by these stringent security regulations. The wireless node has restricted access to the resources that are available and is vulnerable to a large number of various types of attacks. If just a few of the sensors are breached, the adversary may only have to steal a considerable number of keys. The confidentiality of the data can be protected using a variety of cryptographic techniques, but doing so requires a sizable amount of computational power in addition to other resources. These issues have become the primary focus of this research project, whose goal is to design a WSN that can provide secure routing.

The most important objective of this research project is to identify methods in which mobile sinks may be used to make wireless sensor networks (WSN) safer. The following is a list of some of the project's other objectives:

- To develop and put into action the implementation of a tiered refuge architecture in order to defend wireless sensor networks (WSNs) from attacks that involve the replication of static nodes.
- The goal of this project is to develop and build a safe and secure localised sensor reprogramming methodology for use in WSNs that include mobile sinks.
- To develop a security system for WSNs that is based on Hermitian matrices and incorporates a mobile sink.
- To develop a safe and reliable protocol for localised sensor reprogramming in wireless sensor networks (WSNs).

The following is a list of the parts that will be included in the proposed paper: the primary objective and problem statement of the proposed work and a literature survey of the existing methodologies are included in Section 2; the methodology and algorithmic description of the proposed work are included in Section 3; the results and discussion of the proposed work are included in Section 4; and Section 5 concludes the proposed work.

## 2. Study of Related Work

According to the findings of a number of investigations, there are a significant number of issues that do not yet have any answers or solutions. This is the case as of right now. According to Ref. [22], the Internet of Things is plagued by a plethora of challenges and issues that have not been satisfactorily handled as of yet and must be overcome before it can be fully realised. A few examples of problems that need to be solved include interoperability, an Internet of Things-based business model in which thousands or millions of devices can be linked in a network, as well as security and privacy challenges such as entities authentication and authorisation, trustworthiness, and end-to-end security. These are just a few examples of the problems that need to be solved. The authors, on the other hand, came to the realisation that efficient security models are essential in order to turn the Internet of Things into a reality. Reference [23] presented an alternative point of view in which they said that there are basically two obstacles to tackle, namely, security challenges and privacy concerns. Reference [23] supplied this other point of view. They have thought about all of these concerns that have no answers and discussed the numerous facets of these issues with one another. During the course of the conversation on the security issues, a few of the following subjects have been brought up: authentication and authorization, key distribution and management, data storage and safe processing, secure data transmission, and protection against denial-of-service attacks. Likewise, when they investigated the challenges created by the topic of privacy, they provided information on the privacy of passive users, privacy options, identity management, and the necessities of enterprises. In their analysis [24], they focused their attention on the several safety concerns that are connected to the implementation of IoT. The authors make it very clear that before the Internet of Things can be implemented on a large scale, there are a number of big barriers and serious issues that need to be addressed. Even before Internet of Things can be fully realised, this must be accomplished. The processing and administration of massive amounts of data to guarantee relevant information and service while also guaranteeing the integrity and confidentiality of data is one example of such a challenge. Another is the management of heterogeneous devices, which can be complicated by limited network capacity. Reference [25] has put out an exhaustive study of the security issues and challenges that are present at each layer of the IoT architecture in order to make their findings public. They have designed a standard architecture for the security of the Internet of Things in order to preserve the privacy and confidentiality of the data that are stored on these devices. Reference [25] provides a method of classification that is one of a kind in order to address the many dangers and challenges that are brought about by the Internet of Things (IoT). This classification is accomplished by making the safeguarding of data the major focus of Internet of Things security, which in turn allows for the achievement of the aforementioned categorisation. The implementation of this technique included conceptualising the Internet of Things as a layered architecture. The attacks are grouped together as four-layer attacks, each of which comprises software, physical, network, and encryption assaults in that order. The weaknesses in the Internet of Things' currently implemented security measures were outlined in Reference [26], along with an overview of those methods. They have provided a security framework so that we may circumvent some of the constraints. They found out how vulnerable the Internet of Things (IoT) was by using a statistic known as the Threat Index (TI), which was constructed on the basis of many characteristics extracted from the IoT ecosystem. This TI, in combination with the index threshold, may be used to give assistance to the IoT provider in the process of gathering information on the current state of the network's security. Sathish and Dhiren investigate the threats to users' data privacy and information security that are presented by the layered architecture of the Internet of Things (IoT). In addition, it was discovered that there are a number of issues that have not yet been fixed, and these issues are tied to the privacy and safety of IoT. These are the issues that the researchers need to focus on in order to construct a secure platform in the near future. According to Reference [27], the Internet of Things (IoT), which is defined by its many various forms of technology, has privacy and security requirements that must be met in order to play a vital role. The authentication of data as well as its secrecy, the management of access within the IoT network, trust and privacy between devices and users, and the implementation of security and privacy standards and rules are some of the requirements that are mentioned in this article. Within this framework, the security needs, in addition to some of the probable hazards, are outlined at each of the four layers of the IoT architecture. Application security, general device security, network security, and communication security are the four areas into which the potential threats may be divided. Reference [28] presented the results of a research that focused on the risks that are related with the Internet of Things (IoT). The authors carried out an in-depth investigation of the many unresolved challenges and worries about data protection that are raised by the Internet of things (IoT). These strategies have to be implemented in order for the infrastructure of the Internet of Things (IoT), applications, security attacks, backdoors, and usage of wireless sensor networks to function well. In addition, the authors did research on the design guidelines that should be taken into account for any security system in order to offer authenticity, integrity, and secrecy. After that, the authors highlighted the utility of the proposed structure by stating that it had the potential to increase the effectiveness of the security defensive ability in IoT [29]. A structure of this kind has the potential to be able to put into effect the items listed below:

- Integration of a trusted module (user, perception, terminal, and agent);
- Reduction in the likelihood of dangers to network safety;
- Resolution of the practical demands of users;
- Trustworthy extension of the functions related to the Internet of Things; and
- Management of the various information security resources. In addition to this, the authors stressed how important it is to do more study in order to address the issues that were brought up about safety.

## 3. Methodology of the Proposed Work

This article takes a high-level look at the MANET automatic address setup process and investigates it from many angles [30]. It examines the various approaches to address setting and then presents an original approach to address setting that protects the MANET against the Sleep Deprivation attack. In addition to that, it offers a summary of these various approaches. An unplanned network that operates on IP and is made up of a collection of mobile and wireless nodes is referred to as a mobile ad hoc network or MANET for short [31]. A MANET is also known by its acronym.

There are two main forms of MANETs that may be identified from one another. These are single-hop and multi-hop MANETs. A network is said to be single-hop if each node in the network is able to have direct connection with each and every other node that is within the same radio range [32]. On the other hand, nodes in a multi-hop network are reliant on other intermediate nodes to transfer the data if the destination node is located outside the radio range of the node in which they are located. This is due to the fact that multi-hop networks have a greater number of nodes than conventional networks. Compared to conventional networks, the sorts of networks in question provide a much higher number of difficulties and impediments [33]. A MANET has what is known as a dynamic topology, which indicates that it is always changing and does so in a way that is difficult to anticipate. In addition to this, the various kinds of nodes that are involved in a MANET each have their own unique link capabilities, and those link capacities themselves may change depending on the connection that is being used [34]. In addition, the performance of a MANET may be substantially affected by the occurrence of frequent disconnections, problems in transmission, erroneous network design, and a huge number of issues over the network's security. As a result of the nodes in an ad hoc network having limited resources, the network itself is composed of devices that are powered by batteries [35]. In addition, the nodes in the network itself have limited resources.

Both the automated setting of IP addresses and intrusion detection systems are essential components of ad hoc networks. Ad hoc networks also need automatic setup of MAC addresses. The process of automatically assigning a conflict-free and unique IP address to every participating node in the MANET without any human intervention or by making use of any centralised DHCP server is referred to as the IP address auto-configuration task. This task must be completed in order for the IP address auto-configuration to be successful. A possible definition for this activity is the task of automatically setting an IP address [36]. Before a new node can begin to actively participate in a MANET network, an IP address that is unique and does not overlap with any of the other addresses already present in the network must first be assigned to it. This ensures that another node in the network will not use the new address. In order for the nodes that make up a MANET to interact with one another, the nodes themselves must first be configured with link-local addresses that are valid inside the MANET. Only then will the nodes be able to communicate with one another. In addition, in order for the nodes to be able to connect with the outside world, it is possible that they will need to declare global routing addresses [37].

MANET nodes, in addition to having auto-configuration, are required to offer a mechanism for calculating the trust value of each other node. This allows for the identification of which nodes are malicious and which are selfish inside the network [38]. Every node in the network is responsible for assessing the reliability of every other node in the network based on its own personal experience as well as the information it has garnered from the nodes that are immediately next to it in the network [39]. If the trust value of this node falls below a specific threshold, it is presumed that the node is malicious or self-serving, and as a result, the node is removed from the trust graph as an entity that should not be relied upon. It is required that a one-of-a-kind identity be assigned to each node, and this identity must subsequently be validated in the way that is deemed acceptable. Fixing the auto-configuration method in such a way that it does not let any nodes to get multiple identities is one of the steps that have to be taken in order to get rid of the misbehaving node. It is necessary that the auto-configuration system be designed in such a way that no node is able to obtain more than one address. This can only be accomplished by developing the system in such a way. If a node has the capacity to get several identities, it will be considerably easier for a hostile node to conduct a Sybil attack or an IP spoofing attack. Both of these types of attacks involve impersonating another network node's IP address. To deceive the trustworthiness evaluation, the adversary may use either each identity in turn or all of them simultaneously, depending on their preference. Because the integrity of the routing system is dependent on the trustworthiness of the nodes, some of the nodes may be malicious and attempt to change the trustworthiness of other nodes that are more trustworthy. This is because the trustworthiness of the nodes is dependent on the integrity of the routing system. In addition to this, if the malicious node is discovered, it may suddenly go offline. After this, it may attempt to rejoin the network using a more recent set of identities in order to launch a new attack. This process may be repeated until the malicious node is successfully removed from the network. When a MANET lacks centralised administration and has a peer-to-peer design, it is impossible to uniquely identify each node in the network. This makes it difficult to track down and eliminate malicious actors.

Because either an IP or a MAC address may be easily spoofed, using either one of these addresses as a credential does not provide any protection against attacks such as Sybil or IP spoofing. This is because both of these addresses can be easily spoofed. The present technologies, in order to appropriately offer a unique identity to a node, either need human intervention or demand a centralized trust module (such as a Trusted Third Party–TTP or DHCP server). In other words, it is not something that can be done automatically. As a consequence of these limits, the performance of the MANET is severely impeded in a number of application contexts, which might cause considerable problems. It is possible to “fingerprint” the hardware of a mobile device by making use of certain distinguishing characteristics that are immutable and cannot be modified. This is one possible way to solve the problem. For example, the “Manufacturer Serial Number” (MSN) of the hard disc drive that is burnt into the hard disc controller in a laptop, as well as the “PDA Serial Number” that is stored in a PDA. Both of these numbers are unique to the device in which they are located. These kinds of immutable properties of a device need to be unique, and they may be used in the process of computing unique identification if they are accurate. These immutable, consistent attributes of the hardware identity of the device may be used as strong credentials, and an IP or MAC address may be tied to them in order to prevent nodes from faking IP or MAC addresses. This is done in order to protect the integrity of the network. This is done to prevent spoofing of IP or MAC addresses, which might have serious consequences.

### 3.1. Problem Definition

This suggested method, known as RNGHID, is intended to defend against two significant DoS assaults, known, respectively, as the Sleep Deprivation attack and the Sybil attack. Both of these assaults are brought on by the ineffective setting of the addresses being used. In this article, we will discuss a brand-new Intrusion Prevention System (IPS) that has been developed specifically for MANETs.

A protocol is suggested to eliminate assaults on MANET that are caused by IP address conflicts. This would be accomplished by dynamically assigning a one-of-a-kind IP address to each and every node that is part of a MANET.

#### 3.1.1. Delegate Entity Attack (DEA)

Delegate Entity Attack (DEA) is a DDoS (Distributed Denial of Service) attack in which an intruder communicates with another node in a way that looks like a genuine node. However, the aim of this communication is to wake the target node out of its energy-saving sleep mode. In EAACK the authors pointed out that an intruder with the aim of making SD of a node got it done by abusing the weakness of the route discovery process of the protocol through malicious route request (RREQ) flooding. This malicious route request flooding can be categorised into two types:

  Malicious RREQ Type 1:
  An intruder broadcasts an RREQ packet to a destination node with IP address that is within the IP address range but the corresponding node does not actually present itself in the network. This forces all the nodes to forward this RREQ because no node will keep the route for this nonexisting destination IP address.
  Malicious RREQ Type 2:
  After broadcasting an RREQ, an attacker will not wait for the ring traversal time; instead, it carries on resending the RREQ for the same destination with higher TTL values. This is a considerable denial of service attack since energy-constrained operations of MANETs are considered to be very important.

#### 3.1.2. Sybil Attack

Every node in a MANET that wants to take part in routing needs to have a unique IP address, through which nodes are identified. Since MANET lacks a central authority to assign or verify the IP address, an intruder illegitimately claims multiple addresses to send RREQ or RREP packets. A Sybil node can either construct a new identity or forge an identity from a genuine node. This is called the Sybil attack. This is an imitation attack in which the attacker can use either a random IP address or the IP address of any other node to make uncertainty in the routing progress, and it also creates the base for some other severe attacks. In a Sybil attack, a malicious node mimics some nonexistent nodes and it will look like a number of malicious nodes combined together. This attack affects auto-configuration as well. In order to prevent these attacks, we must ensure that every node joining the MANET must be assigned–one and only one‖ IP address.

### 3.2. Random Number Generator with Hierarchical Intrusion Detection System (RNGHID)

This section describes the RNGHID (a Random Number Generator with Hierarchical Intrusion Detection System) scheme in detail. RNGHID consists of four major parts, namely, MSN, IP_COMPUTE, allotment table, and MNA (Malicious Node Alert) message.

**Figure 1:**
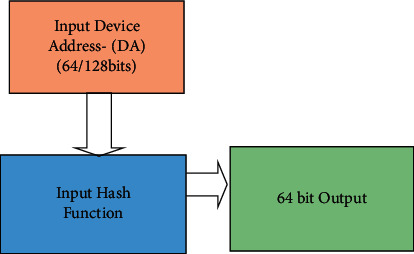
Block diagram of the proposed random number generator with hierarchical intrusion detection system.

There are numerous auto-configuration mechanisms already proposed in the literature. Even though most of the existing algorithms assume the security of MANET, the improper assignment of IP addresses for the MANET nodes causes the above said types of attacks. In this article a protocol is proposed, which dynamically assigns an IP address to every node coming into the network with a strategy that is to say–every node must be given one and only one IP address‖. As the first step we consider an independent ad hoc network functioning itself, later it can be extended to ad hoc networks participating in the Internet of Things. Our proposed protocol is based on a simplified cryptographic hash function that accepts a device address (DA) and produces output as a 16-bit value. The input may be a 48-bit Ethernet MAC address, a Bluetooth address, a UWB address or a 64-bit Zigbee address, or any other unique identification of a node that wants to participate in the MANET.  Figure 1 shows the proposed approach.

### 3.3. System Model

This model-independent, ad hoc network is created from one node as the origin and then the remaining nodes join the network one by one. The nodes are free to move everywhere and can join or leave the network at any point in time. Hence, a dynamic topology will be created and it can predict the size of the network. It can define the *lifetime* of the MANET as the period between the first node configuring itself with an IP address and all the nodes that have been left out or switched off.

#### 3.3.1. Protocol Design Goals

For assigning an IP address, the protocol should meet the following criteria:

- Every node in the MANET should be given a unique IP address, i.e., at any given point of time more than one node should not be assigned the same IP address. Furthermore, a node should be given only one address during the lifetime of the MANET.
- The protocol should ensure that if a node leaves the MANET and attempts to rejoin after some time (within the lifetime of the MANET), the IP address assigned to the node remains the same as previously assigned and should not be changed at any cost.
- The protocol should handle the situations of network partitioning and merging. When two different MANETs merge, there are possibilities that two or more nodes have the same IP address. The protocol must be capable of spotting and correcting such duplicate identities.
- The protocol should ensure that only authorised nodes are configured and approved to access the network resources.

#### 3.3.2. The RNGHID Algorithm

This section presents the proposed algorithm for dynamic IP configuration. A device address (DA) like Ethernet MAC address, Bluetooth Address, or any other equivalent identification (Hardware address of Zigbee or UWB protocol) can be used to calculate the IP address of a MANET node by a simplified cryptographic hash function. This algorithm is called a Random Number Generator with Hierarchical Intrusion Detection System *(RNGHID) algorithm*. The technique proposed here makes every node a *provider* to a new node *N*_new_. Thus, all the nodes are talented to calculate and assign IP addresses from the physical address of the new node *N*_new_ and so *N*_new_ can acquire an address just from its neighbors. Each *provider* computes a unique IP address for a new host *N*_new_ from the physical address given by *N*_new_. Thus, broadcasting a request message for searching a server or for DAD is not required.

There are three phases in our proposed RNGHID algorithm. In phase I, the first node *N*_first_ of the MANET configures itself and becomes a provider. In phase II, a new node *N*_new_ makes a request for IP address and in phase III the provider node computes and offers a new IP address to *N*_new_. Algorithms for the first two phases are given in Algorithm 1, and an algorithm for the third phase is given in Algorithm 2, respectively.

**Figure 2:**
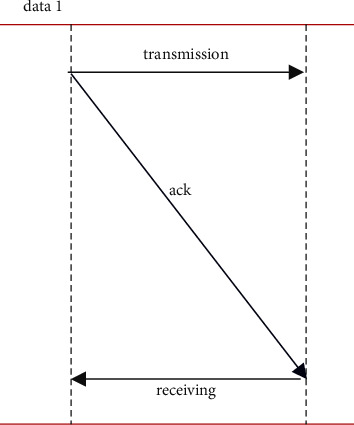
Packet transmission in the proposed algorithm.

(a) *Address allocation for the first node*: When a new node *N*_new_ wants to join a MANET, the proposed RNGHID algorithm broadcasts an IPREQ (Request for IP address) message to its neighbors and it waits for a certain period of time to receive a reply from any one of its neighbors. This message contains its Device Address (DA) as an identifier of the host *N*_new_. The reply message will be a MSNREQ (Request for Manufacturer Serial Number) message. If no MSNREQ message is received, the new node *N*_new_ computes its IP address all by itself by calling the IP_COMPUTE function by supplying its own MAC address as a parameter of the function. The function IP_COMPUTE calculates and returns the host id portion (3^rd^ and 4^th^ octets) of the IP address. In this case the node *N*_new_ becomes the first node *N*_first_ of the MANET and it will be the IP provider for the next newly joining node.
(b) *Address Configuration for the new node*: Assume that a MANET already exists and a new node N_new_ wants to join the MANET and broadcast IPREQ message. Our RNGHID algorithm uses a cryptographic hash function IP_COMPUTE which accepts a Device Address of size 48/64 bits and it generates unique 16-bit value. This 16-bit value will be used in the place of *x*.*x* in the above said IP address.
Figure 2 represents the packet transmission in the proposed work. In order to overcome the fake DA issue, such as the duplicate MAC address problem, it is needed to have one more parameter, which uniquely identifies a node in a network. In general, any ARP and/or RARP packet contains only MAC addresses of the source and destination hosts for unique identification. However, a hidden parameter that exists in every node is its Manufacturer Serial Number (MSN), which is a unique number available in all types of nodes.
The MSN can be read through special commands in various operating systems. In the proposed algorithm, MSN is combined with Device Address which ensures the unique identification of a node in the network. The conversation between *N*_new_ and the provider node will have the following steps:
  1. Node *N*_new_ broadcasts IPREQ message (Contains its Device Address)
  2. Provider node will receive this message and ask for MSN of *N*_new._ (Sends the MSNRREQ message)
  3. *N*_new_ sends back the MSNREP message which contains the Manufacturer Serial Number (MSN) of its own.
  4. Provider node accepts and checks whether an IP address is already assigned for this node.
  5. If already assigned then the provider will send back the same IP address to the *N*_new_ without calling IP_COMPUTE function.
  6. If not (a new MSN is found) then the provider calls the IP_COMPUTE function by supplying the MAC address of *N*_new_ as a parameter to the function to generate the IP address and returns the same to *N*_new_.
  7. *N*_new_ will configure to the IP address and send back the confirmation message IPACK to the provider.
Keeping this in mind IP_COMPUTE is designed: MAC address of a mobile device which wants to participate in the MANET will be read and its length is calculated. In general, the MAC address is 48 bits long in Ethernet, Bluetooth, and UWB technologies whereas 64 bits long in zigbee protocol. Hence in this function, 48 bit addresses are padded with 0's to make them to 64 bits long before further processing. The four binary constants C1, C2, C3, and C4 which are used to avoid collisions while hashing. The entire string is broken up into 4 blocks, namely, *A*, *B*, *C*, and *D*.
On receiving the IPACK message from the new node *N*_new_, the provider node maps the MAC address and the MSN of *N*_new_ with the IP address and it is encapsulated in an MSN_MAC_IP message then broadcasted to all nodes within its radio range (including *N*_new_). After receiving this message from the provider, *N*_new_ performs a final check on the configuration parameters. The neighbors after receiving the MSN_MAC_IP message broadcasts it to its neighbors and so on such that the whole of the MANET receives it within a short time. The nodes then update their allotment table by inserting the MSN, MAC address, and the IP address of the new host *N*_new_ in the allotment table entry.
Partitioning and Merging of Network: In the RNGHID address allocation process that has been presented, more than one node will not have the same IP address at any one moment in time. Because of the fluid and unpredictability of the MANET network, partitioning in the network takes place. However, due to the use of MSN in the RNGHID protocol that has been suggested, there will not be any conflicts of address inside the network even in the event that the network is partitioned. According to what was said before, the IP address of a particular node will not be given to any other host even if that particular node disconnects from the MANET or goes offline while the MANET is in operation. Because each node has the potential to generate one-of-a-kind IP addresses for a new node, there will not be any address conflicts even if the two networks that were previously split come back together. In addition, the suggested procedure is able to properly deal with the following scenario:
  - A node that has been turned off or removed from the network (in most cases, a malicious node) will be automatically detected if it attempts to rejoin the network while it is still active during its lifespan.
  - The MANET may divide into many networks, and then those networks can subsequently combine without any address collisions occurring.
  - Two MANETs that have been separately setup and have distinct net IDs may join together without there being any IP address collisions.
  - Constructing the Allotment Table and Keeping It Up to Date. In the RNGHID scheme that has been presented, the allotment table plays an essential function and has to be kept up to date whenever a new node is added to the network. Each node in the MANET is responsible for maintaining an allotment table, which is where the MSN, MAC address, and IP address of each node are recorded. Following the generation of an IP address by the service provider for the new host Nnew, an allotment table is constructed for the host. In a MANET, every node that takes on the role of provider is responsible for updating its allotment table by adding the new host's MSN, MAC address, and IP address.
(c) *MNA (Malicious Node Alert) message*: The MNA scheme is designed to prevent malicious RREQ message from an attacker. A RREQ is initiated by a source node which has data packets to be sent to a destination. This route request is flooded throughout the MANET to find the route for the destination by its IP address. Each node, upon receiving a RREQ packet, rebroadcasts the packet to its neighbors until the destination is found. In our RNGHID scheme, every node keeps and updates the allotment table, which contains all other nodes' IP addresses which are genuine. Consider a scenario where a malicious node tries to flood a malicious RREQ packet which contains the destination that does not actually exist in the network. Every node that receives this request finds that no such IP address exists in their allotment table and marks the sender as a suspicious node, then RREQ will be refused. The node encounters a suspicious RREQ, initialises its mis_count (malicious node counter) with the IP address of the suspicious node. An MNA message is generated consisting of the suspicious IP address and the mis_count and the same is broadcasted to every other node in the radio range. All other nodes which receive the MNA message rebroadcast it to other nodes, in such a way that the entire network will be alerted in a short time. Suppose if the same malicious node leaves the network and tries to rejoin later, it must seek for an IP address by providing its MAC address and the MSN. As per the RNGHID algorithm, the same IP address will be provided to the node which rejoins. If the node once again tries to broadcast a similar malicious RREQ, it will be denied and the miscount is incremented by one. If the miscount reaches a threshold value, then the suspicious node will be confirmed as a malicious node and will not be allowed to enter the MANET once again. Then the threshold is set as a minimum as required. In this way, the sleep deprivation attack can be effectively prevented.

### 3.4. Performance Evaluation

The experiments are conducted and they analyse the performance of the proposed idea using NS2 simulator. These experiments are focused on collecting the results of address allocation Latency, Communication overhead, and the number/type of messages exchanged by our protocol, at the same time preventing the attacks due to improper IP address assignments. The work is used to find two types of attacks: DDoS attacks and Delegate Entity attacks.attacks, namely, (i) Delegate Entity Attack by uniquely assigning IP addresses in a different way. The RNGHID protocol is tested by using the following parameters:

- Random waypoint mobility model.
- Network area is 1000 m × 1000 m.
- Nodes move with a maximum speed of 25 m/second.
- The routing protocol used was the Ad hoc On-demand Distance Vector (AODV).
- Transmission range of the node is 100 m.
- Data link layer was IEEE 802.11 for all the nodes.
- The number of nodes in the network is 100
- Routing Protocol: AODV

The proposed protocol is tested and compared with the other well-known protocols for address assignment, and for Intrusion Detection.  Table 1 gives the performance comparison of RNGHID with other existing schemes. The following metrics are taken for evaluating the performance:

 
*Distributed process*: In a MANET, dedicating a particular node as a configuration server is not possible due to mobility, low transmission range, and limited battery power. Therefore, the protocol should be distributed among all the nodes in the MANET.
 
*Complexity*: Considering the limited computation power and memory capacity of mobile nodes, an attempt is made to reduce the complexity of algorithms.
 
*Communication overhead*: The solution has two parts. First is the IP address configuration, which requires only neighbor node communication. The second part, the MNA part, is used to alert the nodes about the entry of the malicious node, which requires broadcasting.
 
*Uniformity*: Since the protocol is distributed among the nodes and a cryptographic hash function is used to generate the IP addresses, the address range is uniformly distributed.
 
*Latency*: Since the protocol involves communication between neighbor nodes for address allocation, it creates only a shorter latency for address allocation.
 
*Scalability*: The scheme for address allocation allows every node as an address provider. Therefore, the number of nodes joining the network is not limited to the address space.

## 4. Experiment Analysis and Results

In this section, a case study with different attack setups and analyses are made to test the proposed RNGHID. It presents the simulation results of these experiments and some significant conclusions from the investigation of the attacks.

**Figure 3:**
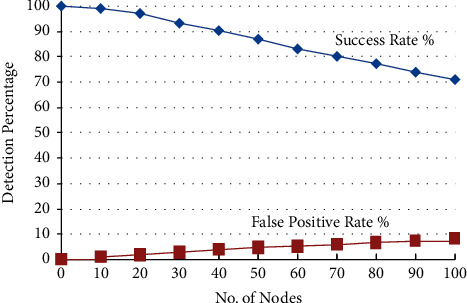
Success and false positive rates of SD attack against the number of nodes.

The first experiment is conducted to test the performance of RNGHID against sleep deprivation attacks using malicious RREQ flooding (MRF) attacks. The chart in Figure 3 shows the success rate (SR) and False Positive Rate (FPR) of RNGHID by accounting for the number of nodes in the MANET with SD attack, whereas Figure 3 illustrates the detection SR and the FPR rate for the Sybil attack. SR here means the rate of correctly spotting the intrusion in the network, identifying the attack type, and then pointing out the node which is triggering the attack.

**Figure 4:**
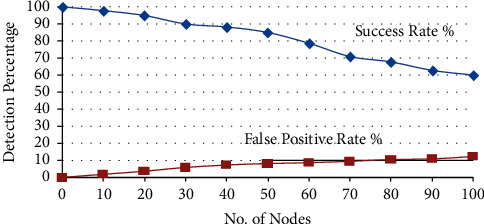
Success and false positive rates of sybil attack.

The term “False Positive Rate” (FPR) refers to the frequency with which normally functioning nodes are incorrectly detected and segregated. In comparison to the SD attack, the graph demonstrates that RNGHID has superior performance in terms of high SR and low FPR rates.  Figure 4 depicts the typical amount of time, measured in milliseconds, required for address allocation while nodes are moving at a variety of speeds.

**Figure 5:**
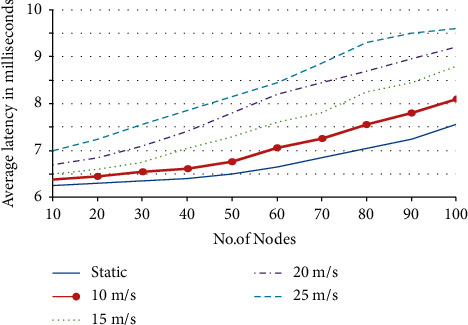
Average address allocation latency in milliseconds.

When the nodes are moving at various speeds, the average amount of protocol overhead, measured in kilobytes, is shown in Figure 5. Figure 5 depicts the typical message overhead, and it demonstrates that the ACPIO technique achieves favorable results when compared with alternative protocols that are already in use.

**Figure 6:**
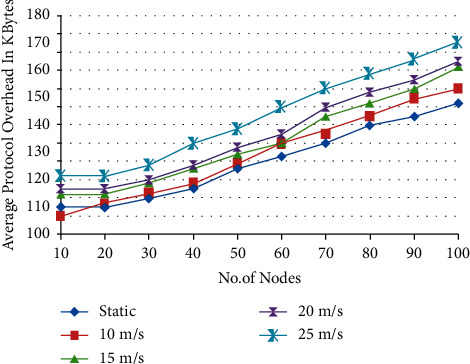
Average protocol overhead.

**Figure 7:**
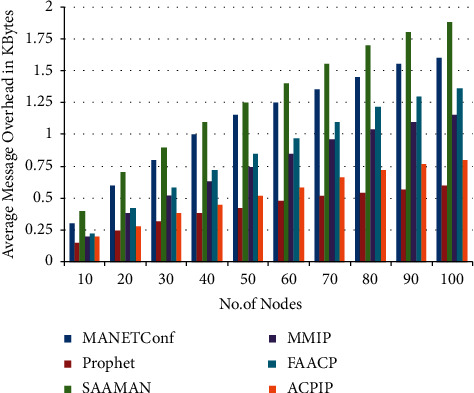
Average message overhead.

Figure 6 represents the average protocol and Figure 7 represents the average message overhead.

## 5. Conclusion

This article presents a unique and innovative approach to intrusion detection and prevention known as an Intrusion Prevention System (IPS). In order for the intrusion prevention system to function properly, each device is given a unique IP address.

MANETs. Reassigning a node's one-of-a-kind address after it has rejoined a MANET is one of the most critical obstacles that must be overcome. When a node in a MANET redistributes its IP address after rejoining the network, this leaves the network open to the possibility of being attacked with a denial of service. The system is responsible for distributing addresses, and thus is very susceptible to communication breakdowns and to MANET being torn apart and then pieced back together again.

In this article, a method is shown that links the Manufacturer Serial Number (MSN) of a node to the IP address that has been allotted to that node. This feature assures that a node inside the MANET will not be able to change its IP address throughout the lifetime of the MANET, even in the event that the node's media access control (MAC) address is changed during that time. Because of this, there is no longer a need for neighbours to transmit regular communications to one another. Each computer that is part of the network plays the part of an address provider in the algorithm. This gives each host the ability to distribute IP addresses to other hosts that join the network. Due to the fact that doing so would result in a substantial reduction in the amount of bandwidth used, it is not essential to flood the MANET with the signalling message that also contains the MNA message. Because the MANET does not flood with any signalling messages other than the MNA message, the amount of bandwidth that is needed and the amount of battery that is consumed are both significantly reduced. The findings of the simulation tests show that the proposed method has an acceptable latency, a low communication overhead, and a singularity in the provision of an IPv4 address while concurrently mitigating DoS attacks in a single MANET. It is possible that in the future support for IPv6 will be added to the technique, which would need further computation. In addition to this, the major concentration of emphasis will be placed on developing an Intrusion Prevention System (IPS) that is capable of protecting MANET in combination with the Internet of Things (IoT).

## Figures and Tables

**Algorithm 1:**
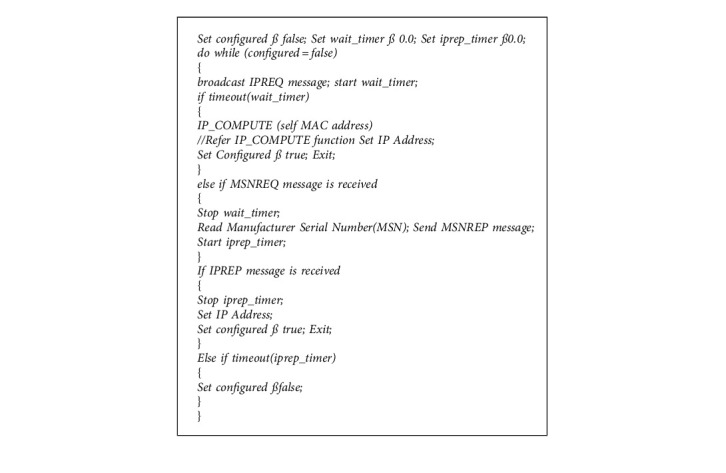
Configuration of *N*_first_ and *N*_new_.

**Algorithm 2:**
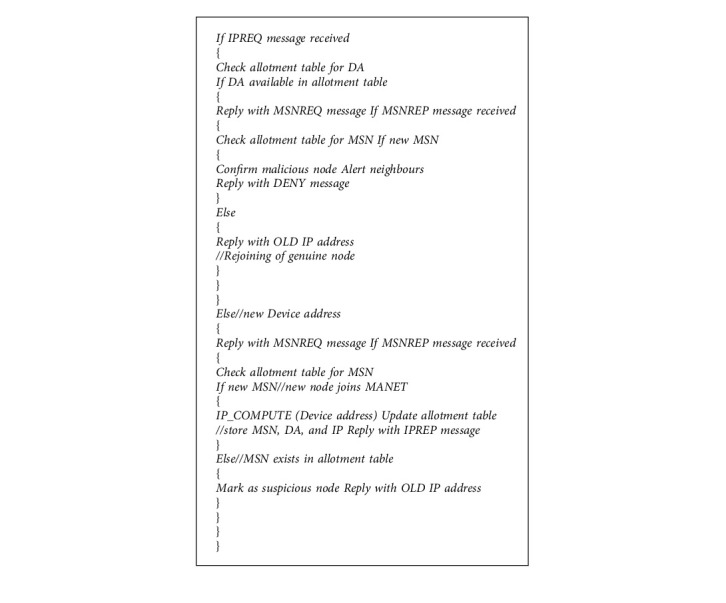
Provider node response.

**Table 1:** Performance comparison of RNGHID with other schemes.

Metrics	MANET conf	Prophet	PrimeDHCP	RNGHID
Complexity	High	Low	Medium	Low
Communication overhead	High	Low	Medium	Medium
Latency	High	Low	Low	Medium
Scalability	No	Yes	No	Yes
Uniqueness	Yes	No	No	Yes
Intrusion attack	No	No	No	Yes

## Data Availability

The data that support the findings of this study are available on request from the corresponding author.
